# Sex matters: retrospective data collection on the date of commencement of ART for HIV-positive women and men in the German KompNet cohort (1991-2009)

**DOI:** 10.1186/1758-2652-13-S4-P20

**Published:** 2010-11-08

**Authors:** I Krznaric, A Wienbreyer, A Zoufaly, P Kreckel, S Esser, T Harrer, K Jansen, C Michalik, S Hertling

**Affiliations:** 1 Praxis Driesener Straße, Berlin, Germany; 2 University Medical Center Hamburg Eppendorf, Infectious Diseases Unit, Hamburg, Germany; 3 Praxis Mehringdamm, Berlin, Germany; 4 University Medical Center Essen, Essen, Germany; 5 University Medical Center Erlangen, Erlangen, Germany; 6 German Komp Net, Bochum, Germany

## Background

In Germany the majority of people living with HIV/AIDS are males and little is known about the characteristics and epidemiology of female HIV patients. The German KompNetKohort is a large, national retro- and prospective, multicenter, HIV-specific cohort, that documents HIV-positive patients since 1991. One important question we wanted to answer is in how much these two populations differ and whether a gender specific treatment approach is needed.

## Methods

A thorough database analysis was conducted and male and female HIV patients of the German KompNetKohort were stratified for baseline characteristics, immunologic status (CD4+ counts, VL, opportunistic infections) before commencing ART, country of origin, way of transmission as well as the initial therapy regimen.

## Results

We analysed 3793 Patients (590 women (15.5%), 3203 men (84.4%)). The median age was 43,6 years for women and 47,64 for men. 137 female (23.3%) and 751 male (32.9%) had CD4- counts < 200 /µl, whereas 451 women and 1529 men showed CD4- counts > 200 CD4/µl ( p< 0.001). The initial therapy regimen was documented for 2216 patients. 215 women and 687 men started with a PI-based regimen, whereas 199 women were treated with an NNRTI and 1314 men ( p< 0.001). Other regimens where used 176 times in women and 1403 in men.

## Conclusion

In the German KompNetCohort men had lower CD4-counts at the time of starting ART. Women were much more frequently treated with PI than men. A reason could be the restrictions in child bearing age for efavirenz and the CD4-cell count for nevirapine use.

It is not clear if the lower CD4-Cellcount at time of starting ART in men is due to late presentation. Further investigations are warranted.

